# The ENTPD5/mt-PCPH oncoprotein is a catalytically inactive member of the ectonucleoside triphosphate diphosphohydrolase family

**DOI:** 10.3892/ijo.2013.2052

**Published:** 2013-08-06

**Authors:** CAITLIN M. MacCARTHY, VICENTE NOTARIO

**Affiliations:** 1 Department of Radiation Medicine, Lombardi Comprehensive Cancer Center, Georgetown University Medical Center, Washington, DC, USA

**Keywords:** cellular energy balance, ENTPD5/mt-PCPH, malignant transformation, nucleoside triphosphate diphosphohydrolase activity, oncoprotein

## Abstract

Expression of the ENTPD5/mt-PCPH onco-protein and overexpression of the normal ENTPD5/PCPH protein contribute to the malignant transformation of diverse mammalian cell types, and PCPH is mutated and/or deregulated in various human tumor types. Expression of PCPH or mt-PCPH caused similar phenotypes, yet the effects promoted by mt-PCPH expression were consistently and substantially greater. ATP depletion and increased stress-resistance are phenotypes commonly associated with PCPH and mt-PCPH expression. It was suggested that the intrinsic nucleoside triphosphate diphosphohydrolase (NTPDase) activity of PCPH and mt-PCPH may be responsible for these phenotypes, but direct supporting evidence remains to be established. Results from experiments designed to test such hypothesis demonstrate that, as expected, mt-PCPH expression in human colorectal carcinoma (CRC) cells decreased their ATP levels and conferred resistance to oxaliplatin, a colorectal cancer-relevant chemotherapeutic agent. Using a combination of site-directed mutagenesis, immunoprecipitation methods, *in vitro* enzyme activity assays and *in situ* enzyme activity determinations in live cells, this report also demonstrates that the mt-PCPH oncoprotein lacks detectable NTPDase activity, indicating that direct ATP cleavage by mt-PCPH did not cause the ATP depletion observed in mt-PCPH-expressing CRC cells. These results strongly suggest that the mt-PCPH oncoprotein may regulate the cellular energy levels and subsequent chemoresistance by an NTPDase-independent mechanism. Understanding possible alternative mechanisms will be essential to devise strategies for the successful treatment of predictably therapeutically resistant tumors expressing either increased PCPH levels or, particularly, the mt-PCPH oncoprotein.

## Introduction

The energy demands on rapidly proliferating cancer cells necessitate major metabolic adjustments that either precede malignant transformation, pushing cells toward carcinogenesis, or come about as adaptations to the stressful environmental conditions to which tumor cells are subjected ( 1 ) . One of the most common metabolic alterations is the acquired ability of a cell to use glycolysis as the primary ATP source even in the presence of oxygen, known as the Warburg effect ( 2 ) . The Warburg effect has been demonstrated in multiple tumor types ( 3 ) and is generally accepted to be necessary for the growth and survival of tumor cells and, hence, glycolytic metabolites and glycolysis regulating enzymes could be used as metabolic tumor markers ( 3 ) . In fact, studies of colon tumor metabolites and key glycolytic enzymes demonstrated the power of these markers in discriminating tumor stage ( 4 ) , location ( 5 ) , malignancy and metastatic ability ( 6 ) .

The nucleoside triphosphate diphosphohydrolases (NTPDases), also referred to as apyrases, make up a family of enzymes responsible for intracellular and extracellular nucleotide metabolism. This well-conserved family of nucleoside phosphate cleaving enzymes has eight known members, which have been shown to impact biological processes such as apoptosis, adhesion, and differentiation ( 7 ) . Knockout studies have further indicated a role for NTPDases in tissue development, cell proliferation, inflammatory response and metabolism ( 8 , 9 ) . NTPDase 5 is distinctive from the other NTPDases as it is the only member described as a protooncoprotein, better recognized as such by the name PCPH. The proto-oncogene and oncogene were discovered when chemical carcinogen treatment of Syrian hamster embryo fibroblasts activated the *ENTPD5/PCPH* gene into its oncogene counterpart ( *mt-PCPH* ) ( 10 ) . Comparison of the transforming and normal sequences revealed a single base pair deletion in the open-reading frame (ORF) of the oncogene. As a consequence of this mutation, the ORF was shifted resulting in a truncated gene product relative to the normal protein ( 11 ) . Although the ENTPD5/PCPH protein has been reported to promote N-glycosylation, cell proliferation and the Warburg effect ( 12 ) , the biological processes in which the PCPH and mt-PCPH proteins participate and their molecular functions in mammalian cells remain unclear.

The transforming activities of the overexpressed normal PCPH and of the mt-PCPH oncoprotein were demonstrated in various cell systems ( 11 , 13 – 15 ) and, accordingly, their expression has been detected in multiple cancers ( 16 – 18 ) . Generally, expression resulted in phenotypic changes shared by PCPH and mt-PCPH yet the effects were consistently and substantially greater in cells expressing mt-PCPH compared to PCPH. Two phenotypes normally associated with PCPH and mt-PCPH expression are depleted cellular ATP levels (13, and unpublished data) and increased resistance to stress ( 11 , 13 – 15 ) . It was demonstrated that reduced ATP levels play a part in the concurrent chemo-resistance observed in mt-PCPH expressing cells. ATP repletion experiments in BALB/3T3 cells expressing mt-PCPH restored their sensitivity to cisplatin treatment ( 14 ) . However, the mechanism by which these proteins decrease cellular ATP levels remains unclear.

It was suggested that the intrinsic NTPDase activity of PCPH ( 19 ) and mt-PCPH ( 20 ) may be responsible for the cleavage of ATP and hence for the concomitant stress-resistance ( 15 ) . Given that ATP is not a favored substrate for hydrolysis by PCPH ( 19 ) , this explanation seemed unlikely. Still, direct evidence linking the NTPDase activity of PCPH or mt-PCPH to ATP hydrolysis in the cell remains to be shown. Data in this report demonstrate that mt-PCPH expression reduced ATP levels and conferred oxaliplatin chemo-resistance in colorectal carcinoma (CRC) cells. Results also demonstrate that the mt-PCPH oncoprotein did not show any detectable NTPDase activity in assays performed *in vitro* under a number of different experimental conditions or when tested *in situ* in living cells, indicating that the ATP depletion observed in mt-PCPH-expressing CRC cells most likely did not result from the direct cleavage of endogenous ATP by mt-PCPH. These findings strongly support the notion that the mt-PCPH oncoprotein is catalytically inactive and imply that it may regulate the cellular ATP content by NTPDase-independent mechanisms. Understanding these alternative mechanisms will be essential to devise strategies for the successful treatment of predictably therapeutically resistant tumors expressing either increased PCPH levels or, particularly, the mt-PCPH oncoprotein.

## Materials and methods

### Reagents

RPMI-1640 medium, antibiotics, and general cell culture reagents were from MediaTech, Inc. (Manassas, VA, USA). Magnetic beads (protein G dynabeads), Novex ^®^ Tris-Glycine Precast gels, Geneticin ^®^ (G418), Optimem ^®^ and Lipofectamine™ 2000 transfection reagents were from Invitrogen (Carlsbad, CA, USA). TrueBlot ^®^ reagents were from eBioscience Inc. (San Diego, CA, USA), and the myc-peptide was from AnaSpec Inc. (Fremont, CA, USA). Nucleotides and other general chemicals were from Sigma-Aldrich (St. Louis, MO, USA). The following antibodies were used: anti-myc-tag (Cell Signaling, Danvers, MA, USA), anti-ENTPD5 (Sigma-Aldrich), and HRP-conjugated secondary antibodies (Cell Signaling). The protease inhibitor cocktail was from Roche Applied Science (Indianapolis, IN, USA).

### Cell culture and transfection

Human CRC HCT116 and prostate cancer PC-3 cells were from the ATCC (Manassas, VA, USA), and human CRC HCT15 cells were from our Cancer Center’s Tissue Culture Shared Resource. Cells were maintained in RPMI-1640 medium containing 10% fetal bovine serum, 100 *μ* g/ml streptomycin, and 100 IU penicillin, at 37°C, 5% CO _2_ and 85% humidity. Hamster *PCPH* (AF084569) and *mt-PCPH* (AF084568) ORFs were cloned into pcDNA3.1 (+)-myc-HisB mammalian expression vectors as described ( 20 ) . Transient and stable transfections were performed as described ( 18 ) . For chemical treatments, equal cell numbers were plated 24 h prior to replacing the medium with chemical- or vehicle-containing medium.

### ATP replenishment

Liposome preparations (Encapsula NanoSciences, Nashville, TN, USA) were composed of phosphatidylcholine, cholesterol and dipalmitoylphosphatidylglycerol at 9:4:0.1 molar ratio, with a 24.6 mg/ml total lipid concentration. The ATP content of ATP-liposomes was determined for each batch before use. For ATP replenishment experiments, actively dividing HCT116 cells transfected with pcDNA, PCPH or mt-PCPH were counted and plated in 12-well dishes at 1.75×10 ^5^ cells per well for ATP analysis or in 96-well dishes at 1×10 ^4^ cells per well for cell viability assays. Following 24 h in culture, the medium was replaced with fresh medium containing 0.5% of either ATP-encapsulated or empty liposomes, continuing the incubation for 8 h. After this treatment, cells were washed thoroughly with PBS and collected for ATP analysis. For chemosensitivity assays, liposome-containing media were removed and cells were washed with PBS and incubated with either vehicle- or oxaliplatin-containing medium for 24 h before processing for cell viability determinations.

### Immunoblot and immunoprecipitation

Western immunoblot analyses were carried out essentially as described ( 14 ) , being performed at least three times for each protein. Lysates for immunoprecipitation (IP) were prepared in modified RIPA buffer (50 mM Tris pH 7.8, 140 mM NaCl, 0.5% sodium deoxycholate, 1% NP-40) containing protease inhibitors. IPs were performed using protein G-conjugated dynabeads according to manufacturer’s recommendations (Invitrogen). Tris-buffered saline (TBS) was used instead of PBS and care was taken to prevent phosphate contamination. For immunoblot analysis of IP samples, bead pellets were boiled in Laemmli sample buffer (LSB) in the presence of β-mercaptoethanol. Myc peptide elution was performed as described ( 21 ) , pooling two incubations with 0.5 *μ* g/ *μ* l of peptide followed by TCA precipitation and resuspension in LSB containing β-mercaptoethanol.

### Nucleotidase assay

NTPDase activity was detected by measuring the release of inorganic phosphate in the presence of nucleotide substrates as described ( 22 ) . Cell lysates were prepared in modified RIPA buffer containing protease inhibitors. Briefly, whole-cell lysates or IP beads were incubated at 37°C with 75 mM Tris-HCl pH 7.5, 10 mM CaCl _2_ , 10 mM MgCl _2_ and 0.1% Triton X-100, with or without substrate. Reactions were stopped by adding malachite green solution (1.2% malachite green, 4% sulfuric acid, 0.2% Tween-20). The optical density was measured at 630 nm and reported in reference to phosphate standards.

### Enzyme cytochemistry

Enzyme activity visualization in cultured cells was performed using a lead phosphate detection method as described ( 23 ) . Transiently transfected cells grown to 70% confluency in 6-well dishes were washed, fixed with 4% paraformaldehyde in TBS solution, permeabilized with 0.1% saponin, and incubated in the reaction solution [20 mM Tris-maleate, 500 mM sucrose, 2 mM MgCl _2_ , 2 mM CaCl _2_ and 2 mM Pb(NO _3_ ) _2_ ] for 30 min with or without 2 mM GDP. Reactions were developed by incubating with 1% ammonium sulfide. Samples were then blocked with 10% normal goat serum and then sequentially incubated with dilutions of anti-myc-tag antibody and a fluorophore-conjugated secondary antibody. Cells were imaged immediately using an Olympus IX71 fluorescence microscope at our Cancer Center’s Microscopy and Imaging Shared Resource. Phase-contrast and fluorescent images collected from 5 random fields were densitometrically analyzed using ImageJ software ( 24 ) .

### ATP measurements

Cells were counted, plated in 6-well dishes (0.5–1×10 ^6^ cells) and collected after 24 h. Cell pellets were resuspended in boiling buffer and incubated at 100°C as described ( 25 ) . ATP measurements were performed on supernatants using a luciferase/luciferin-based kit (Invitrogen). Values are presented relative to an ATP standard curve as the average ATP concentrations corrected by total protein estimates of the supernatants performed using the BCA system. Measurements were taken from at least two cell passages, in triplicate.

### Cell viability assays

Cells were counted, plated in 96-well plates (1–30×10 ^3^ cells), and after 24 h the medium was replaced with fresh medium with or without oxaliplatin. Treated and untreated cells were incubated again for 24 or 48 h, and cell viability was measured using the Cell TiterGlo system (Promega, Madison, WI, USA), according to manufacturer’s recommendations. Luminescence was detected using a Berthold Microlumat Plus LV 96V luminometer from our Cancer Center’s Genomics and Epigenomics Shared Resource.

### Statistical analysis

Data are presented as mean ± standard deviation (SD; error bars) for a given experimental group or observation. One-way ANOVA were performed to determine the statistical significance of differences between experimental groups. p≤0.05 was regarded as significant.

## Results

### ATP levels and chemo-resistance in CRC cells expressing mt-PCPH

**Figure 1.**
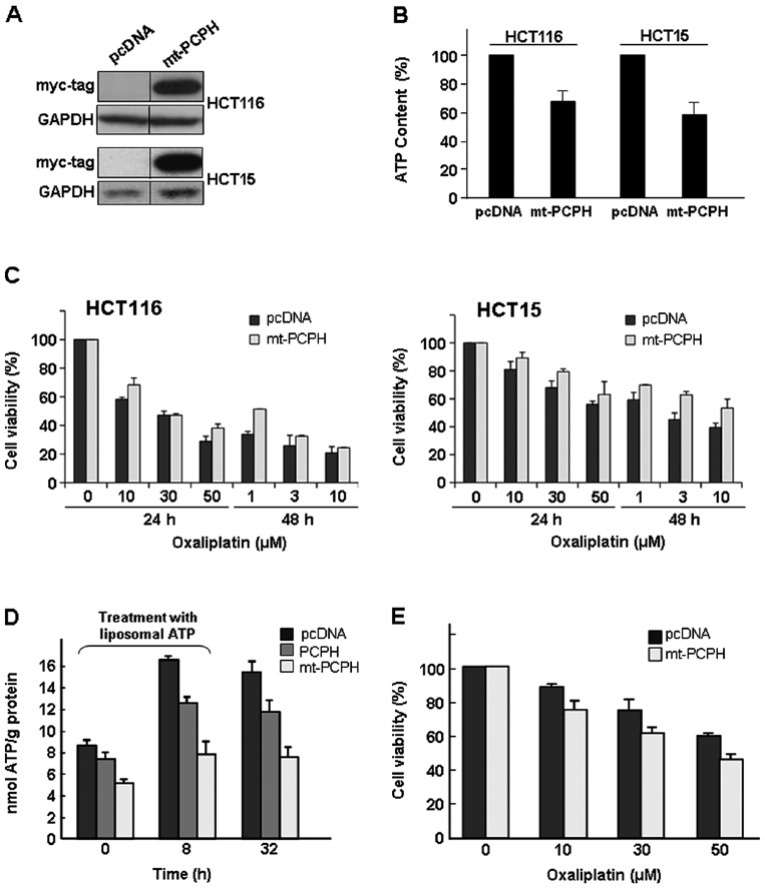
Effect of mt-PCPH on the ATP levels and the chemo-response of colon carcinoma cells. (A) Western blot analysis of lysates from HCT116 and HCT15 cells stably transfected with empty vector (pcDNA) or myc-tagged *mt-PCPH* ; GAPDH was used as loading control. (B) ATP levels of cells from panel A. ATP measurements were normalized using ATP standard curves and lysate total protein concentration. Measurements were performed in triplicate from three cell passages (n=3). (C) Cell viability of cells in panel A in response to oxaliplatin treatment. Experiments were repeated for two cell passages in triplicate (n=2). (D) ATP levels of empty vector (pcDNA), *PCPH* or *mt-PCPH* transfected HCT116 cells were determined (n=2) at the beginning (time zero) and the end (8 h) of the treatment with empty or ATP-encapsulated liposomes, and again 24 h after completion of the treatment, removal of liposomes and incubation in fresh liposome-free medium (32 h). (E) Cell viability of HCT116 cells stably transfected with empty vector (pcDNA) or *mt-PCPH* from (D), treated with oxaliplatin (n=2) after exposure to empty or ATP-containing liposomes.

To understand the relationship between mt-PCPH catalytic activity and the reduced ATP levels and enhanced chemo-resistance conferred by mt-PCPH, ATP levels were determined in extracts of HCT116 and HCT15 CRC cells, which express low endogenous levels of PCPH or mt-PCPH, after stable transfection with empty vector (pcDNA) or myc-tagged *mt-PCPH* ( Fig. 1A ). HCT116 cells expressing mt-PCPH (HCT116/mt-PCPH) exhibited ATP levels around 28% lower than those of pcDNA-transfected (HCT116/pcDNA) cells ( Fig. 1B ). Similarly, ATP levels were nearly 40% lower in HCT15/mt-PCPH cells than in the HCT15/pcDNA controls ( Fig. 1B ). In comparison to the action of mt-PCPH, PCPH expression had little effect on the ATP levels (∼5% decrease) in either HCT116 or HCT15 cells.

To establish the effect of mt-PCPH on their chemo-response, HCT116/mt-PCPH and HCT15/mt-PCPH cells were treated with several oxaliplatin concentrations for 24 and 48 h. HCT116/mt-PCPH cells were more resistant to oxaliplatin (about 25% after 24 h and 35% after 48 h) than control cells ( Fig. 1C , left panel). Similarly, HCT15/mt-PCPH cells were also more resistant to oxaliplatin (about 14% after 24 h and 31% after 48 h) than control cells ( Fig. 1C , right panel).

To test the importance of reduced ATP levels in mt-PCPH-induced chemo-resistance, the endogenous ATP levels of HCT116/pcDNA, HCT116/PCPH and HCT116/mt-PCPH cells were replenished by treatment with ATP-encapsulated liposomes for 8 h, and cells were then analyzed for their response to oxaliplatin exposure. HCT116/pcDNA cells treated with ATP-encapsulated liposomes showed a nearly 2-fold increase in ATP levels relative to their ATP content prior to the treatment ( Fig. 1D ). The magnitude of the increase in ATP content of HCT116/mt-PCPH cells after incubation with ATP-encapsulated liposomes was smaller (∼1.5-fold) than in control HCT116/pcDNA cells ( Fig. 1D ), but reached ATP levels in the same range as those found in HCT116/pcDNA and HCT116/PCPH cells prior to liposome treatment ( Fig. 1D , t=0). The increase in ATP levels rendered the HCT116/mt-PCPH cells more sensitive to oxaliplatin than control cells ( Fig. 1E ), reversing their typical drug response and suggesting that ATP plays a role in mt-PCPH-induced cellular chemo-resistance.

### Generation of NTPDase-deficient PCPH and mt-PCPH mutants

**Figure 2.**
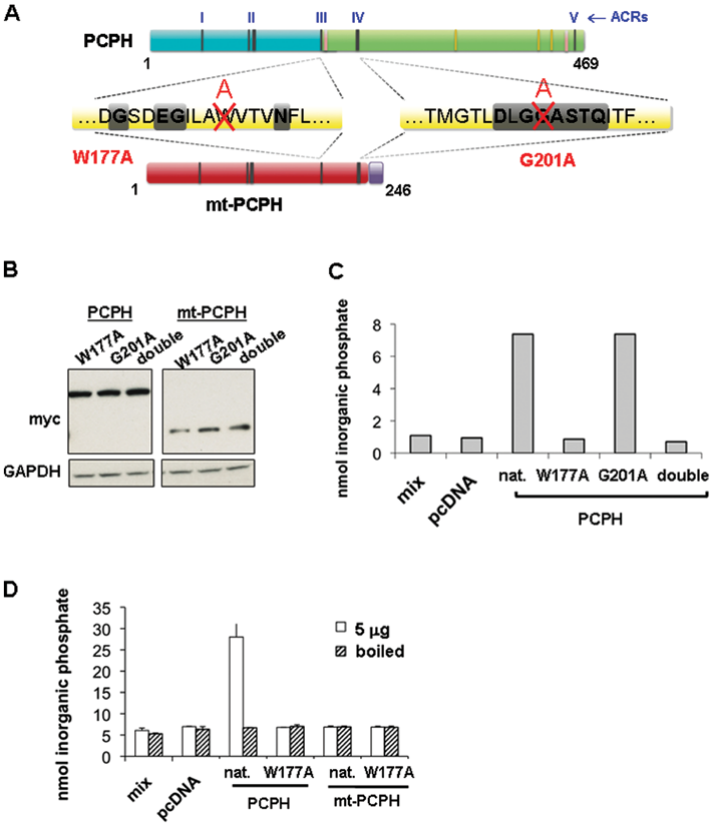
Design, expression and GDPase activity of apyrase mutants. (A) Schematic illustrating the mutagenesis of tryptophan 177 to alanine (W177A) and glycine 201 to alanine (G201A) to generate putative inactive NTPDase variants of PCPH or mt-PCPH; ACRs, apyrase conserved regions. (B) Western blot analysis of cell lysates from HCT116 cells 48 h after transient transfection with W177A, G201A or W177A+G201A (double) mutated PCPH or mt-PCPH constructs. (C) GDPase activity assay of cell lysates from HCT116 cells transiently transfected with native (nat.), W177A, G201A or W177A+G201A (double) PCPH mutants. Assays included 5 *μ* g of total protein and reaction mix containing 2 mM GDP, and were incubated at 37°C for 5 min. Reactions without protein (mix) and lysates from empty vector-transfected cells (pcDNA) were included as controls. (D) GDPase assays of 5 *μ* g of cell lysates from empty vector (pcDNA), native (nat.) or W1771A mutated PCPH or mt-PCPH-transfected HCT116 cells. Reactions executed as described for (C) were run in duplicate, and one control set was boiled prior to incubation with GDP.

As the NTPDase activity of PCPH and mt-PCPH enzyme activity had been previously reported ( 11 , 19 , 20 , 26 – 28 ) , to determine if the enzyme activity of mt-PCPH was directly responsible for the cleavage of ATP and, therefore, for the observed decrease in endogenous ATP levels, we generated inactive apyrase mutants of both PCPH and mt-PCPH using a site-directed mutagenesis approach. The tryptophan at position 177 (W177) and the glycine at position 201 (G201) were selected for mutagenesis ( Fig. 2A ) on the basis of successful inactivating mutations of related NTPDases previously reported in the literature ( 22 , 29 , 30 ) . The mutagenesis process converted both amino acid residues into alanine, generating the W177A and G201A single mutants as well as the double mutant of PCPH or mt-PCPH. All constructs were first sequenced to confirm the successful generation of the expected inactivating mutations, then transiently transfected into HCT116 cells, and whole extracts of the transfected cells were assayed for NTPDase activity using GDP as the substrate (GDPase) as previously described ( 31 ) . GDP cleavage was monitored by the detection of released inorganic phosphate using a malachite green colorometric assay ( 32 ) . Expression of the PCPH and mt-PCPH NTPDase mutants in HCT116 cells was confirmed by immunoblot analysis of cell lysates probed for the myc-tag ( Fig. 2B ). In comparison with extracts of HCT116/PCPH cells, GDPase activity assays of extracts from cells expressing mutated PCPH showed that the W177A mutation completely abolished the GPDase activity, whereas the G201A mutation had no effect on the activity ( Fig. 2C ). Accordingly, the variant containing both mutations (double) did not show GDPase activity. The GDPase activity of cell lysates from HCT116/PCPH cells was lost when boiled prior to incubation with GDP ( Fig. 2D ). However, when the GDPase activity of the native PCPH and the PCPH/W177A apyrase mutant was compared with that of native mt-PCPH and mt-PCPH/W177A, it was revealed, as expected, that lysates prepared from cells containing mt-PCPH/W177A did not show any activity. In addition, most surprisingly, results showed that lysates of cells expressing native mt-PCPH had no detectable GDPase activity either ( Fig. 2D ).

### NTPDase activity of PCPH and mt-PCPH expressed in mammalian cells

**Figure 3.**
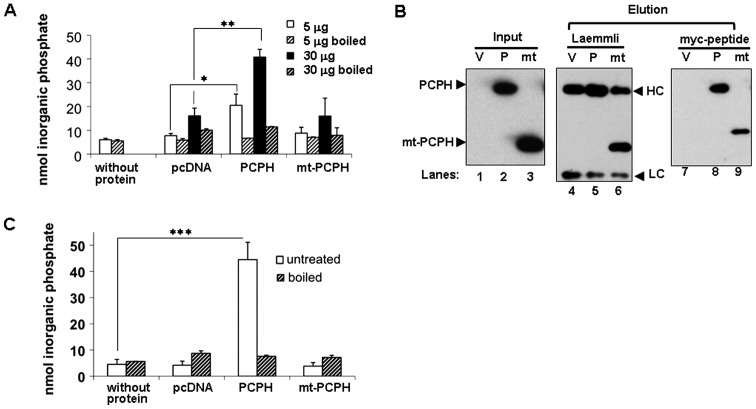
*
In vitro* NTPDase activity of PCPH and mt-PCPH expressed in HCT116 cells. (A) NTPDase assay of lysates from cells transiently transfected with empty vector (pcDNA), PCPH or mt-PCPH; duplicate lysates were boiled or left untreated, incubated with GDP and released phosphate was detected by a malachite green colorometric assay. Each transfection was tested twice and experiments repeated three times (n=3). (B) Western blot analysis of input (20%) and eluates from myc-tag IPs of HCT116 cells carried out 48 h after transient transfection with empty vector (V), *PCPH* (P) or *mt-PCPH* (mt). Proteins eluted using LSB and the myc peptide. Immunoglobulin heavy (HC) and light chains (LC) are detected on blots of IPs eluted with LSB. (C) NTPDase assays were performed as previously described on IPs from C. One way ANOVA ^*^ p≤0.05, ^**^ p≤0.005, ^***^ p≤0.0005.

As the ATP depletion triggered by mt-PCPH expression in CRC and other cell types was attributed to its NTPDase activity, it became important to establish an enzyme activity baseline for both PCPH and mt-PCPH in CRC cells by measuring their NTPDase activity *in vitro* . Lysates from transiently transfected HCT116 cells expressing myc-tagged PCPH or mt-PCPH at comparable levels were incubated with GDP, as described above. Cell extracts containing PCPH showed significant NTPDase activity relative to controls ( Fig. 3A ), for two different total protein concentrations (p≤0.05 for 5 *μ* g and p≤0.005 for 30 *μ* g), which remained at background levels when samples were boiled prior to incubation with the substrate. However, boiled and non-boiled lysates from mt-PCPH-expressing cells yielded phosphate signals indistinguishable from the background levels detected in the corresponding controls ( Fig. 3A ). Similar results were observed from NTPDase activity assays of whole cell lysates from *PCPH* and *mt-PCPH* transfected PC-3 and HCT15 cells (data not shown), indicating that the apparent lack of NTPDase activity by mt-PCPH was not a cell type-specific observation. The fact that negative results were obtained also when GDPase assays were performed with extracts from HCT116 cells expressing an untagged *mt-PCPH* construct ( 20 ) confirmed that the presence of the myc-tag in the mt-PCPH protein did not create a steric hindrance capable of preventing its NTPDase activity.

**Figure 4.**
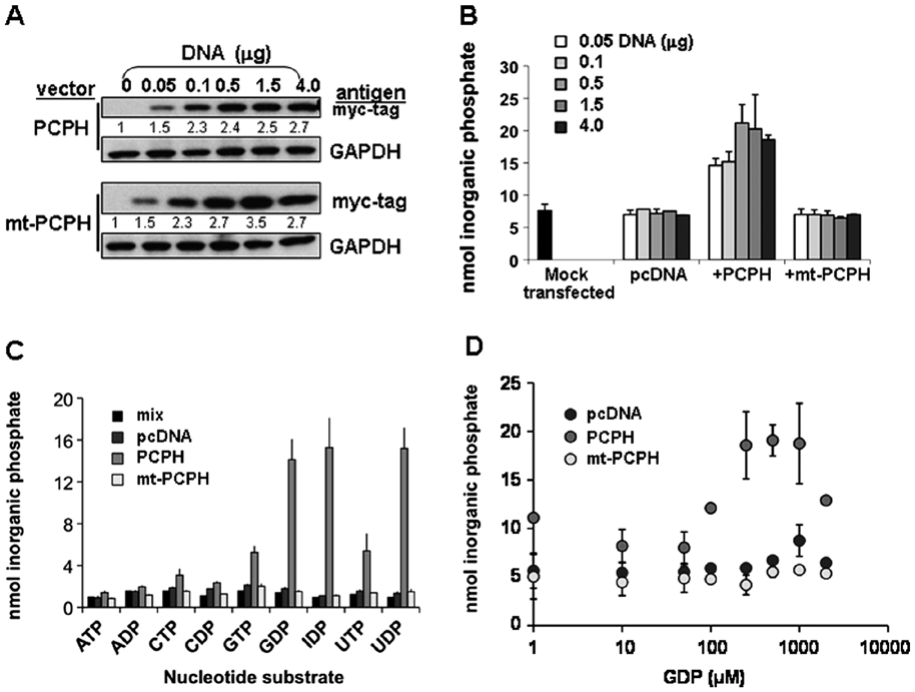
Effect of protein expression level and substrate nature and concentration on the catalytic activity of PCPH and mt-PCPH. (A) Western blot analysis of lysates prepared from HCT116 cells 48 h after being transiently transfected with increasing amounts of *PCPH* or *mt-PCPH* DNA probed for the myc-tag, using GAPDH as the loading control. Relative densitometric values are provided below the blot images. (B) NTPDase activity in lysates (5 *μ* g total protein) of cells described for panel A. Transfections were performed on cells at two different passages (n=2). (C) NTPDase activity of the lysates (5 *μ* g total protein) from HCT116 cells described in Fig. 3 on the indicated NDPs and NTPs. (D) NTPDase activity assays of the same lysates over a range of GDP concentrations (1 *μ* M to 2 mM). Cells were transfected at two different passages and the samples were run in duplicate (n=2).

To establish that the NTPDase activity observed in cell lysates came specifically from the exogenously expressed proteins, IPs were carried out using the myc-tag and the activity of the proteins pulled down was tested on GDP. To confirm their presence of PCPH and mt-PCPH in IPs, proteins were eluted from the beads under reducing and denaturing conditions ( Fig. 3B , lanes 4–6). As under these conditions the antibody heavy chain co-migrated with PCPH, precipitated proteins were also eluted using the myc peptide. Both PCPH and mt-PCPH were detectable in immunoblots from myc peptide-eluted IPs ( Fig. 3B , lanes 7–9). Relative to controls, PCPH IPs showed a significant (p≤0.0005) NTPDase activity ( Fig. 3C ), which was not detectable when IP beads were boiled prior to the assays. On the contrary, boiled and non-boiled mt-PCPH IPs showed no detectable NTPDase activity. To confirm that the differences in NTPDase activity between PCPH and mt-PCPH were independent of their expression levels, activity assays were done using lysates from cells transfected with different amounts of the PCPH or mt-PCPH expression vectors and, therefore, having different PCPH or mt-PCPH contents ( Fig. 4A ). Samples with up to 3.5-fold greater mt-PCPH levels ( Fig. 4A , lower panel) showed NTPDase activity similar to those with low or no mt-PCPH ( Fig. 4B ), whereas the NTPDase activity of PCPH correlated with protein levels in cells transfected with up to 1.5 *μ* g DNA. These data suggested that PCPH does have NTPDase activity *in vitro* , while mt-PCPH may either have severely deregulated activity or lack NTPDase activity. Because differences in primary sequence between PCPH and mt-PCPH could influence the kinetic properties of mt-PCPH enzyme activity, NTPDase assays were performed using different substrates and substrate concentrations. The preferred PCPH substrates ( Fig. 4C ), were in agreement with previous reports ( 19 , 26 , 27 ) , but enzymatic activity remained undetectable in all assays performed with mt-PCPH ( Fig. 4D ).

**Figure 5.**
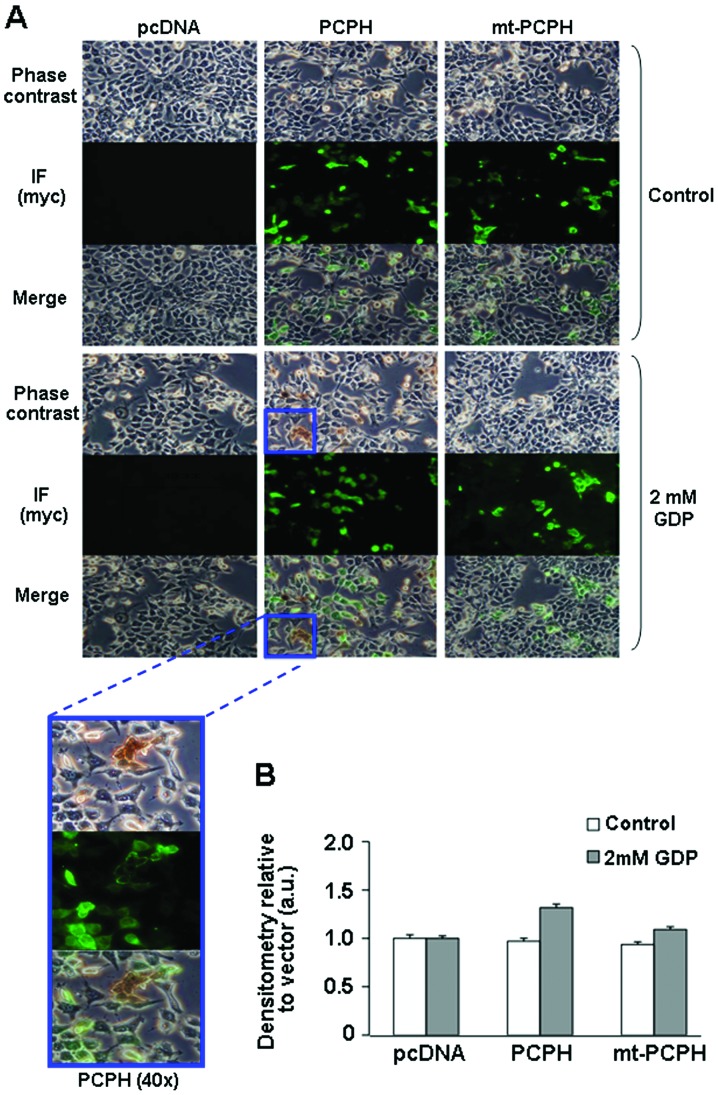
*
In situ* NTPDase activity of PCPH and mt-PCPH expressed in HCT116 cells. (A) Phase contrast images of GDPase activity (brown) and fluorescence (IF) images (×20) of myc-tagged protein expression (green) in HCT116 cells 48 h after transfection with empty vector, *PCPH* or *mt-PCPH* . Transfections were executed on two cell passages (n=2) and transfected cells were tested in duplicate. The blue box shows a ×40 magnification of a field of *PCPH* transfected cells. (B) Densitometry of *in situ* NTPDase activity.

Because it seemed possible that the intrinsic NTPDase activity of the mt-PCPH protein might be destroyed during the process of preparation of the cell lysates, NTPDase activity assays were also performed *in situ* , in actively proliferating HCT116 cells, by combining the use of a phosphate detection method that yields a brown precipitate visible under the microscope, and indirect immunofluorescence to confirm whether the precipitate forms on cells expressing either PCPH or mt-PCPH ( Fig. 5A , IF panels). No precipitate formation was observed in control cultures to which no GDP was added ( Fig. 5A , top panels). In the presence of GDP, subpopulations of cells containing the brown precipitate could be observed in cultures of cells expressing PCPH ( Fig. 5A , middle and magnified panels), but not in cultures of cells expressing mt-PCPH or in the pcDNA-transfected control cells ( Fig. 5A , side panels). Quantification of *in situ* NTPDase activity demonstrated that PCPH, but not mt-PCPH expressing cells, had significant activity compared to vector-transfected controls ( Fig. 5B ). These data reiterated the notion that mt-PCPH most likely lacks NTPDase activity, strongly suggesting that its intrinsic NTPDase activity is not the functional determinant of the ATP depletion and chemo-resistance phenotypes associated with mt-PCPH-induced transformation.

## Discussion

Acquisition of stress resistance has been reported as an important component of the transforming activity of overexpressed PCPH and of the mt-PCPH oncoprotein in different cell systems ( 11 , 13 – 15 ) . It was suggested that such pro-survival role resulted from PCPH and, particularly, mt-PCPH hydrolyzing ATP and consequently depleting cellular energy levels ( 13 , 14 ) . However, the direct evidence linking the mt-PCPH NTPDase activity to ATP depletion and induction of stress resistance was lacking. This report represents the first description of mt-PCPH-induced chemoresistance and ATP depletion in CRC cells, and demonstrates the importance of the endogenous ATP levels in determining the response of mt-PCPH expressing cells to chemotherapy.

The NTPDase activity, *in vitro* and *in situ* , of PCPH and mt-PCPH expressed in human cells is also described for the first time. Surprisingly, results showed that the NTPDase activity of mt-PCPH was undetectable regardless of the cell line, protein expression level, and substrate nature and concentrations tested. These data do not agree with previous studies demonstrating NTPDase activity for recombinant mt-PCPH in non-human expression systems ( 20 ) , and do not support the notion that mt-PCPH catalyzed ATP hydrolysis. This discrepancy is likely due to protein folding, dimerization ability and/or processing differences in the expression systems used, as neither rabbit reticulocyte *in vitro* transcription/translation ( 33 ) nor bacterial expression systems perform post-translational modifications (PTMs) of the type that NTPDase family members are known to undergo, such as palmitoylation, acylation, phosphorylation and glycosylation ( 7 ) . In fact, glycosylation sites are conserved across the NTPDase family ( 34 ) and, accordingly, PCPH glycosylation was demonstrated ( 26 ) . However, NTPDase assays in systems where glycosylation does not occur, was inhibited or removed showing that PCPH glycosylation was not necessary for *in vitro* enzyme activity, although it influenced multimer formation and protein solubility ( 19 ) . The absence of glycosylation or other PMTs in non-human systems ( 20 ) may have caused the observed differences in NTPDase activity by altering native protein processing. However, processing may also influence PCPH and mt-PCPH NTPDase activity in mammalian cells as differential PCPH and mt-PCPH expression was demonstrated for different species, tissues and cell types ( 17 , 35 ) .

Our findings agree with some structural features of mt-PCPH. Fig. 2A shows the N-terminus as conserved within the NTPDase family, including most apyrase conserved regions (ACRs I–IV). PCPH and mt-PCPH also share N-terminal amino acid sequence up to residue 213, after which *PCPH* encodes 256 amino acids and *mt-PCPH* only 33 non-conserved residues ( Fig. 2A ). Due to this truncation, mt-PCPH lacks the cofactor coordinating residue within ACRV, four conserved cysteine residues presumably involved in intra-protein disulfide bonds ( 36 ) , and all residues comprising the substrate-binding pocket ( 37 ) , strongly suggesting that the loss of these conserved features may affect the tertiary structure and enzyme function of mt-PCPH. Other C-terminally truncated NTPDase isoforms have been reported also to be inactive ( 38 , 39 ) .

The crystal structures for PCPH or mt-PCPH have not been resolved, but NTPDase catalytic clefts and structural properties are conserved across the family and are highly homologous to other nucleotide hydrolyzing enzyme families ( 37 ) and, therefore, can be used to aid structural predictions. Using the reported crystal structure of ENTPD2 as a reference for the likely similar structure of PCPH, and not including the C-terminal tail of mt-PCPH, it seems that mt-PCPH would lack nearly the entire second structural domain, which comprises one of the lobes shaping the catalytic cleft and described as containing key active site amino acids and structural features involved in catalysis by other family members. Therefore, although most of the ACRs essential for NTPDase activity are retained in mt-PCPH, the fact that it is missing important catalytic determinants strongly supports the overall soundness of our conclusion that the mt-PCPH oncoprotein is a catalytically inactive member of the NTPDase family of proteins. As indicated in Fig. 2A , the differences in primary sequence, domain organization and structural predictions between mt-PCPH and PCPH could result in an mt-PCPH conformation potentially exposing reactive residues that would otherwise be protected by the second lobular domain present only in PCPH. Consequently, we hypothesize that mt-PCPH might act through protein-protein interactions rather than by way of NTPDase activity.

The metabolic needs of cancer cells are different from those of healthy cells. Cells adapt to the harsh conditions of the tumor microenvironment and metabolic demands for cancer cell survival and growth. Changes in ATP levels subsequent to mt-PCPH expression may be one of these adaptations. In tumors, mt-PCPH expression may be turned on in response to harsh tumor conditions, as endogenous mt-PCPH expression was shown to be stress-induced ( 11 ) , and may increase with tumor progression. Indeed, it was shown that PCPH and mt-PCPH expression was increased in breast ( 17 ) , prostate ( 18 ) , and testicular ( 40 ) tumors compared to normal tissues and PCPH has been described as a biomarker for colon tumors ( 41 ) . It seems that mt-PCPH expression may be not only a powerful reporter for the metabolic shift in tumor cells but also a possible predictor for tumor chemotherapy responses. Understanding the NTPDase-independent mechanisms by which mt-PCPH modifies cellular energy levels and chemo-response may be critical to devise strategies for the successful treatment of predictably therapeutically resistant tumors expressing the mt-PCPH oncoprotein.

## Abbreviations:

ACR: apyrase conserved region;
CRC: colorectal carcinoma;
IP: immunoprecipitation;
LSB: Laemmli sample buffer;
NTPDase: nucleoside triphosphate diphosphohydrolase;
ORF: open reading frame;
PTM: post-translational modification;
TBS: Tris-buffered saline;
TCA: trichloroacetic acid
